# Correction for Kim et al., “Self-assembling Gn head ferritin nanoparticle vaccine provides full protection from lethal challenge of *Dabie bandavirus* in aged ferrets”

**DOI:** 10.1128/mbio.01430-25

**Published:** 2025-06-27

**Authors:** Dokyun Kim, Eunha Kim, Semi Kim, Youseung Chung, Chih-Jen Lai, Inho Cha, Sung-Dong Cho, Yunseo Choi, Xinghong Dai, Stephanie Kim, Seokmin Kang, Mi-Jeong Kwak, Ziyi Liu, Younho Choi, Su-Hyung Park, Young Ki Choi, Jae U. Jung

## AUTHOR CORRECTION

Volume 14, no. 5, e01868-23, 2023, https://doi.org/10.1128/mbio.01868-23. Page 17, Acknowledgments line 6: “AI171443” should be removed.

