# Supplementary figures and images for: Self-assembling Gn head ferritin nanoparticle vaccine provides full protection from lethal challenge of Dabie bandavirus in aged ferrets
Source: mBio. 2023 Sep 15;14(5):e01868-23. doi: 10.1128/mbio.01868-23 (PMC10653821; doi:10.1128/mbio.01868-23)

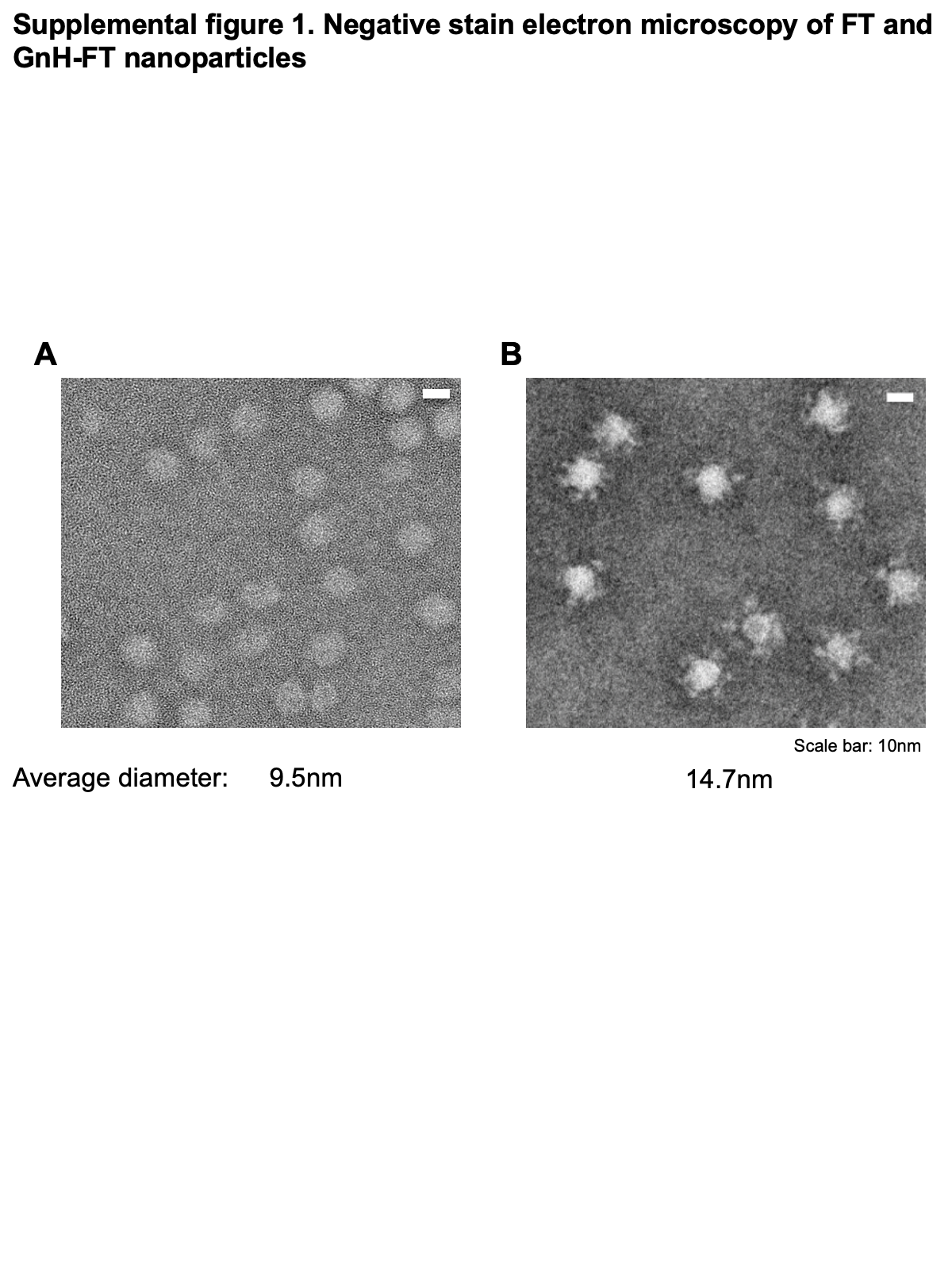

Supplement: Figure S1 — Structural analysis of FT and GnH-FT nanoparticles. [file mbio.01868-23-s0001.tif]

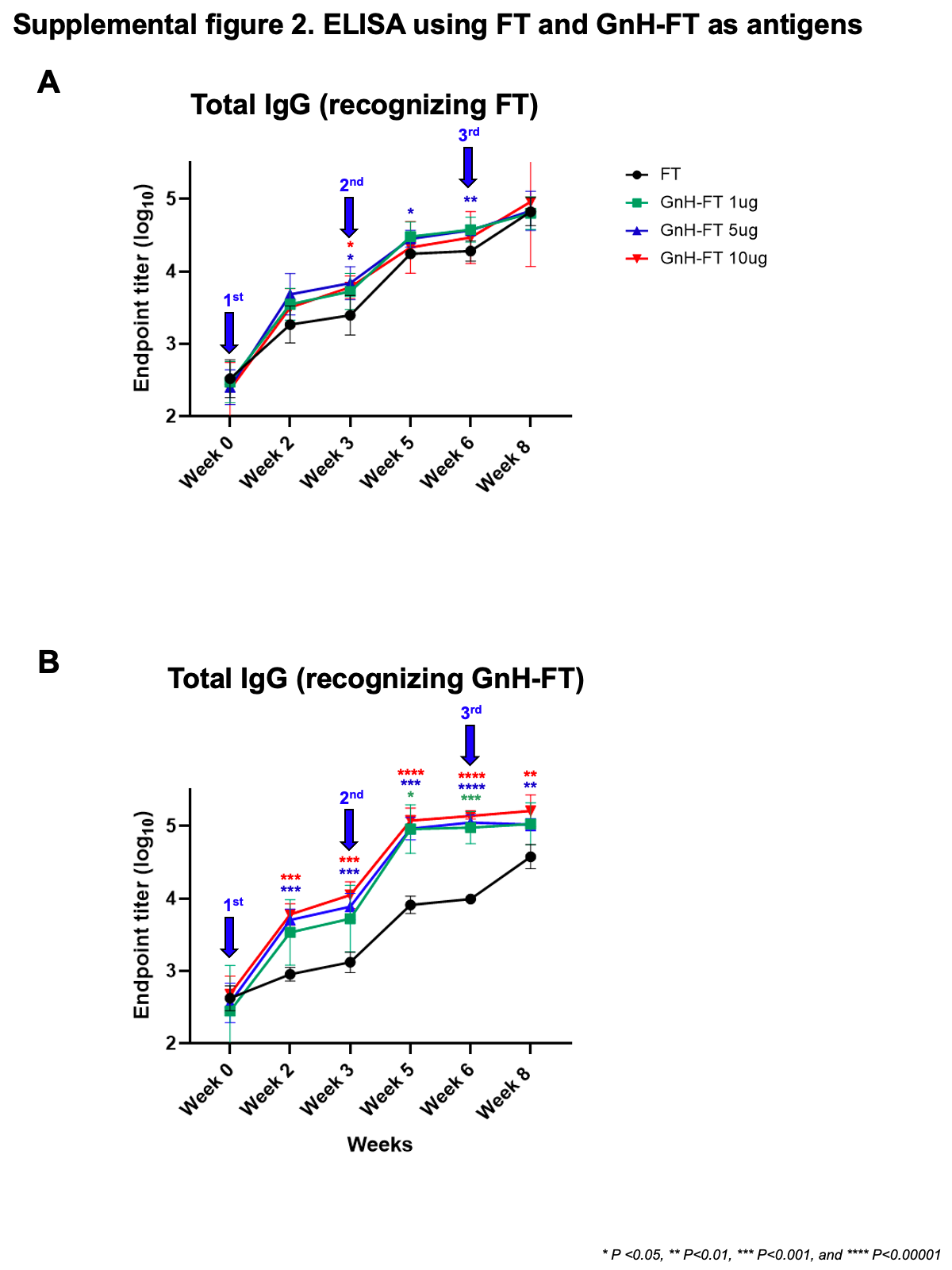

Supplement: Figure S2 — Antibody response against FT and GnH-FT as antigens in immunized mice. [file mbio.01868-23-s0002.tif]
