# Supplementary material for: Self-assembling Gn head ferritin nanoparticle vaccine provides full protection from lethal challenge of Dabie bandavirus in aged ferrets
Source: mBio. 2023 Sep 15;14(5):e01868-23. doi: 10.1128/mbio.01868-23 (PMC10653821; doi:10.1128/mbio.01868-23)
Supplement: Supplemental legends — Legend for Figures S1 and S2. [file mbio.01868-23-s0003.docx]

**Fig S1. Structural analysis of FT and GnH-FT nanoparticles**

(A, B) Negatively stained scanning electron microscopy (EM) of FT (A) and GnH-FT (B) nanoparticles. Average diameters of the nanoparticles were calculated from 50 randomly selected nanoparticles’ diameters.

**Fig S2. Antibody response against FT and GnH-FT as antigens in immunized mice**

(A, B) Experiment from Fig 3B was repeated using FT (A) and GnH-FT (B) as antigens to test for total IgG response against FT and GnH-FT. Total IgG against FT stands for off-target antibody response which targets FT, instead of GnH, as the primary target of the immune response. The asterisks represent statistical significance of endpoint titer from mice immunized with GnH-FT-nanoparticle to mice immunized with FT evaluated with one-way ANOVA with Dunnett multiple comparison test.

*P < 0.05, **P < 0.01, ***P < 0.001 and ****P < 0.00001.
